# Virulent systemic feline calicivirus infection: a case report and first description in Ireland

**DOI:** 10.1186/s13620-024-00262-3

**Published:** 2024-02-09

**Authors:** Antoine A. Duclos, Pedro J. Guzmán Ramos, Carmel T. Mooney

**Affiliations:** Small Animal Clinical Studies, School of Veterinary Medicine, University College Dublin, Belfield, Dublin 4, Ireland

**Keywords:** Feline, Virulent systemic calicivirus, Virology, Reverse-transcriptase polymerase-chain reaction

## Abstract

**Background:**

Virulent systemic feline calicivirus (VS-FCV) infection is an emerging disease. It is distinct from classic oronasal calicivirus infection as it manifests with unique systemic signs including severe cutaneous ulcerations, limb oedema, and high mortality, even in adequately vaccinated cats. Devastating epizootic outbreaks with hospital-acquired infections have been described in the United States, the United Kingdom, continental Europe and Australia with up to 54 cats affected in one outbreak and a mortality rate of up to 86%. This highly contagious and potentially fatal disease has not yet been reported in Ireland.

**Case presentation:**

An 11-month-old male neutered vaccinated domestic shorthair cat was presented with a 10-day history of lethargy, decreased appetite and progressively worsening pitting oedema in all four limbs. The signs were first noted after another kitten from a high-density cat shelter was introduced in to the household. Additional physical examination findings included marked pyrexia, and lingual and cutaneous ulcers. Virulent systemic feline calicivirus was diagnosed based on compatible history and clinical signs, exclusion of other causes, and calicivirus isolation by RT-PCR both in blood and oropharyngeal samples. Negative calicivirus RT-PCR in blood following resolution of the clinical signs further supported the diagnosis.

**Conclusion:**

This case represents the first known case of VS-FCV infection in Ireland. Given the severity of the clinical signs, and the high risk for epizootic outbreaks, Irish veterinarians should be aware of the disease to ensure prompt diagnosis and implementation of adequate preventive measures, in order to limit the threat that this disease represents for the wider cat population and particularly given the risk of hospital-acquired VS-FCV infection. Virulent systemic calicivirus should be suspected in cats with pyrexia of unknown origin, oedema or ulceration affecting the limbs or the face, and exposure to rescue cats from high-density households.

## Background

Feline calicivirus (FCV) is a small, single-stranded RNA virus belonging to the *Caliciviridae* family [1]. Feline calicivirus is widespread in the general cat population, with prevalence ranging from 2.5% in households with less than 4 cats, up to 90% in high-density cat colonies [2]. This high prevalence is explained by the ability of feline calicivirus to persist asymptomatically in the retropharyngeal area of some cats [2, 3]. Feline calicivirus has been shown to be able to evade the host’s immune response through different mechanisms in healthy carrier cats [4], allowing them to become lifelong shedders [5]. Cats are infected through the nasal, oral or conjunctival routes [4]. The infection can be direct, from cat to cat, or indirect via fomites and possibly aerosols [6].

The classic form of the disease is usually characterized by acute, self-limiting upper respiratory tract signs, and less commonly a lameness syndrome or severe pneumonia [4, 5, 7]. Feline calicivirus has also been associated with chronic gingivostomatitis [8].

Due to the viral characteristics (lack of proof-reading and low fidelity), the virus has an important genetic plasticity, which allows it to mutate quickly [7]. The high prevalence combined with the ubiquitous distribution of the virus in the feline population, further increases the likelihood of recombination events and mutations [4]. Thus, there is a high potential for emergence of strains with increased pathogenicity and systemic tropism [9]. Different mutated strains have already been isolated and reported [10, 11].

In the past 25 years, a virulent systemic form of feline calicivirus has been reported in the United States, continental Europe, the United Kingdom, and Australia: virulent systemic feline calicivirus (VS-FCV) [12–19]. It is known to affect cats that have been appropriately vaccinated against feline calicivirus infection [7]. These highly-contagious strains resulting in devastating nosocomial infections have been reported with one single cat suspected of having infected nine other cats in one study [18], as well as outbreaks affecting up to 54 cats [14].

Feline calicivirus binds to a receptor that is located at the tight junctions of endothelial and epithelial cells [7]. This receptor regulates the integrity and permeability of the cell layers [7]. Their disruption leads to oral ulceration in the classic form, and cutaneous ulceration in the virulent systemic form. Vasculitis also plays a role in the pathogenesis of the latter form, and notably participates in the development of cutaneous lesions, oedema, and additional systemic consequences [20]. Although the exact pathogenesis of VS-FCV infection remains poorly understood, it likely involves different cell tropism allowing involvement of visceral organs and systemic involvement and more rapid growth compared to less virulent strains [1, 7, 21]. The vascular damage identified on histopathology are likely immune-mediated and potentially involving local cytokine modulation [21].

The mortality rate for VS-FCV ranges from 22 to 86% depending on the outbreak [19]. Survivors have complete resolution of clinical signs, usually within 7–10 days [12, 16], although duration of clinical signs for up to 40 days have been reported [13, 19].

Whilst highly contagious, basic environmental management (disinfection with sodium hypochlorite, potassium peroxymonodysulfate, chlorine dioxide and commercial products approved for calicivirus inactivation) and isolation measures are effective in preventing spread to other cats [22, 23].

Infection with VS-FCV has not yet been reported in Ireland. However, calicivirus is prevalent in the feline population on the island of Ireland [24]. High-density shelters are present in the country, providing a possible reservoir for emerging mutated calicivirus strains with potential for virulent systemic calicivirus emergence.

This case report provides information on the first endemic case of non-epizootic VS-FCV infection in a cat from Ireland.

### Case presentation

An 11-month-old male neutered domestic shorthair cat was presented with a 10-day history of lethargy, decreased appetite, pyrexia and progressively worsening pitting oedema affecting all four limbs.

The cat was indoor-outdoor, and up-to-date regarding routine vaccination and parasite treatment. It had never travelled outside Ireland. A few days prior to the onset of the clinical signs, a 3-month-old kitten had been adopted from a shelter. The kitten was reported to be free of clinical signs.

The first documented clinical sign in the cat was self-limiting non-weight-bearing lameness of the left pelvic limb. Investigations performed by the referring veterinarian identified mild leukocytosis (18.67 (reference interval 6–18) × 10^9^/L) with neutrophilia (16.8 (reference interval 2.5–12.5) × 10^9^/L) and moderate-to-marked thrombocytopenia (57 (reference interval 180–550) × 10^9^/L) likely related to platelet clumping (adequate platelets on blood smear examination). Enzyme-linked immunosorbent assay (ELISA) for feline immunodeficiency virus (FIV) antibody and feline leukaemia virus (FeLV) antigen were negative. Treatment with cefovecin and non-steroidal anti-inflammatory drugs (NSAID) resulted in a mild transient improvement of the clinical signs. However, there was progressive pitting oedema affecting all four limbs and persistent pyrexia, prompting referral.

**Fig. 1:**
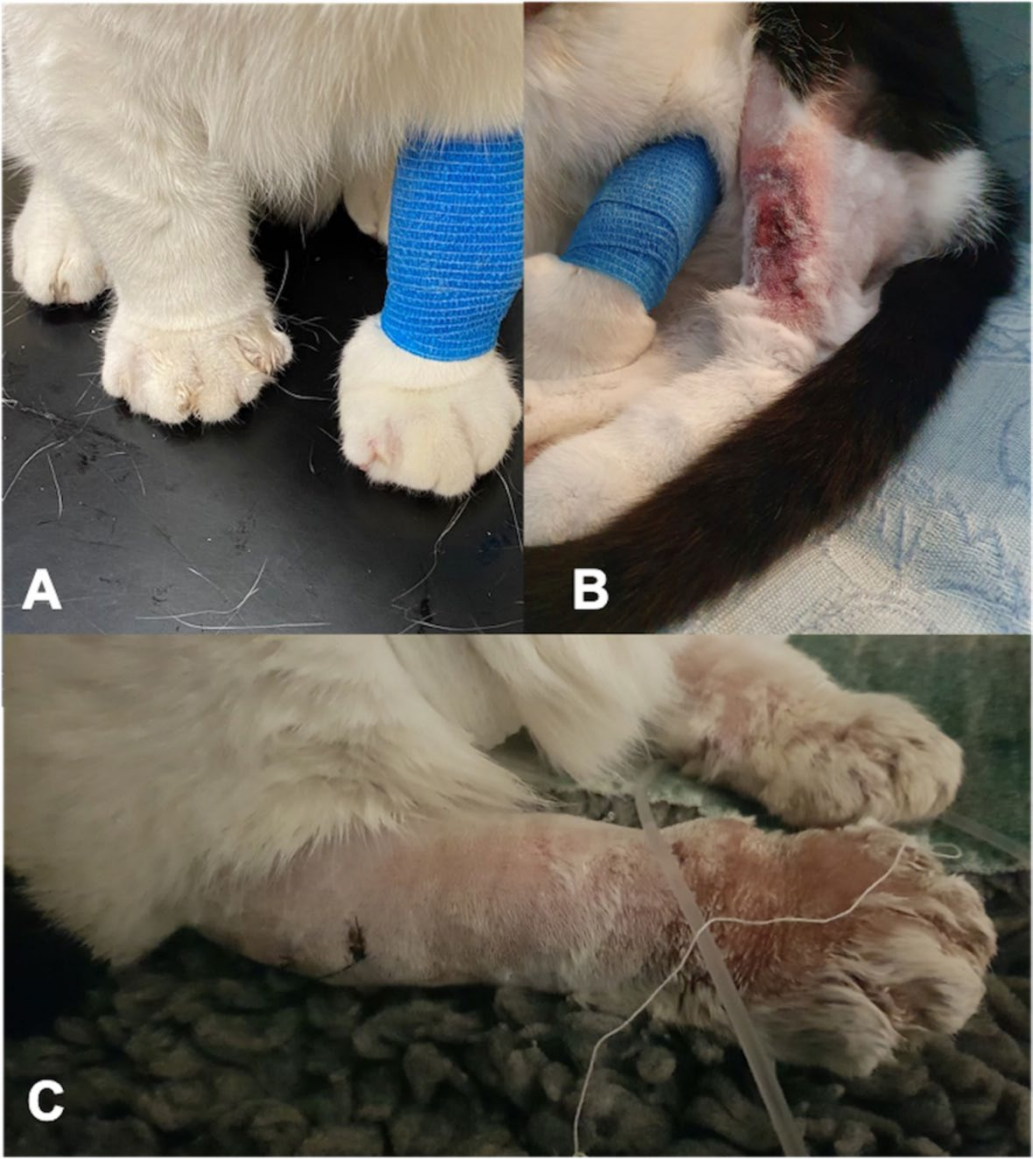
Photograph of the swelling of both thoracic limbs on presentation (**A**), cutaneous ulceration the tibial cranial area of the left pelvic limb (**B**) and exudative lesion after clipping of the right pelvic limb (**C**)

**Fig. 2:**
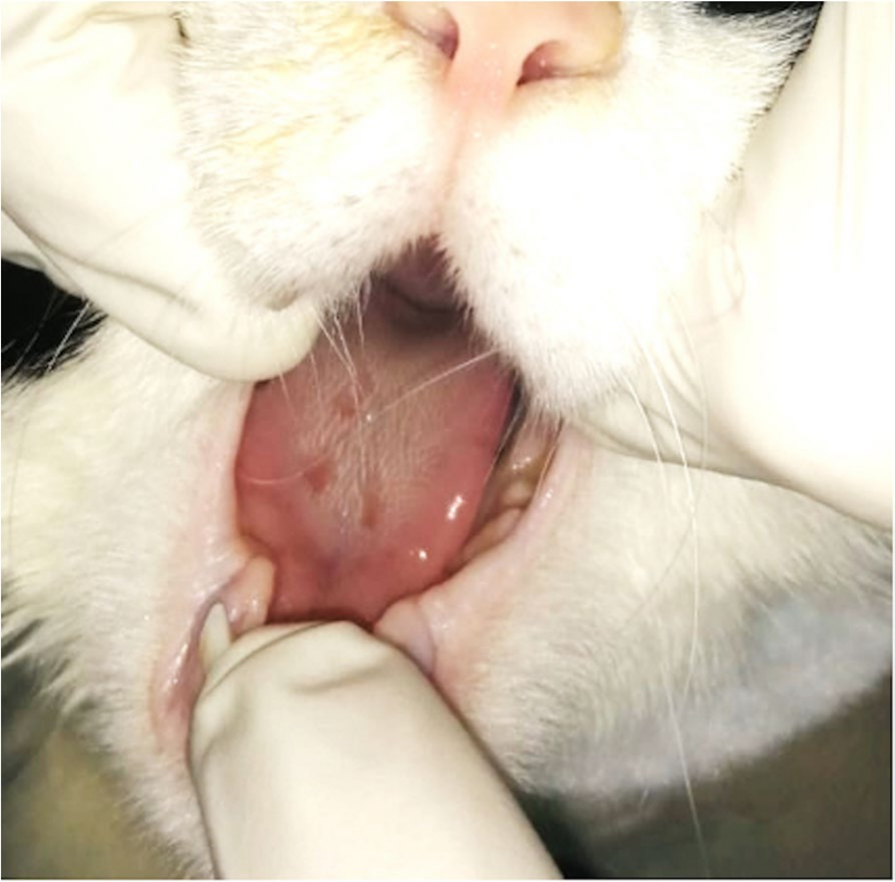
Photograph of the lingual erosions and ulcerations on presentation

Upon referral, physical examination identified increased rectal temperature (40.2 °C). There was severe pitting oedema in all four limbs, with an exudative and ulcerative cutaneous lesion (2 cm diameter) on the cranio-distal aspect of the left pelvic limb (Fig. 1). Buccal examination identified 3 superficial erosions on the dorsal aspect of the tongue (Fig. 2).

The problem list included non-antimicrobial responsive progressively worsening diffuse pitting edema in all-four limbs with cutaneous ulcerations, lingual ulcers and moderate-to-marked pyrexia.

The main differential diagnoses for pyrexia, considering the age and the history, included infectious or inflammatory causes, and less likely neoplasia. The ulcerative lesions in the tongue could be caused by infectious conditions including calicivirus and herpesvirus infections. Mechanical or toxic causes (e.g., foreign body, uraemia, caustic agent) were considered less likely given the subacute worsening clinical progression, the presence of systemic signs and the prior laboratory results. Inflammatory and neoplastic conditions were considered less likely but not ruled out.

The oedema in all-four limbs could have been caused by increased vascular permeability, increased hydrostatic pressure (e.g., right-sided congestive heart failure, venous thromboembolism), decreased oncotic pressure (e.g., hypoalbuminaemia), or diffuse lymphatic disorders (e.g., lymphangitis, lymphoedema). Given the presence of cutaneous and lingual ulcerations, increased vascular permeability related to diffuse vasculopathy was suspected. Differentials included primary inflammatory vasculitis or secondary vasculitis (infectious with bacterial sepsis, mycobacteria, virulent systemic calicivirus, drug-induced, insect bite-induced).

The cat was hospitalized in the isolation unit with barrier nursing and adequate disinfection given the potential risk of virulent systemic calicivirus or mycobacterial infection. Management included intravenous fluid therapy and analgesia (buprenorphine, 15 ug/kg four times daily) which was facilitated by the placement of a venous central line. Peripheral venous access was not possible given the marked oedema affecting all limbs.

Follow-up haematology identified persistent leukocytosis (29.37 reference interval 6–18) × 10^9^/L) with neutrophilia (27.75 (reference interval 2.5–12.5) × 10^9^/L) and mild lymphopenia (0.92 (reference interval 1.5–7) × 10^9^/L) consistent with non-specific inflammation, infection or a stress leukogram. Biochemistry was largely unremarkable. Thoracic radiographs and abdominal ultrasound were performed to investigate for an underlying trigger or an infectious focus. These examinations were unremarkable. As an infectious aetiology was suspected, multimodal testing was performed. Blood cultures were negative. Culture of the exudative cutaneous lesion on the pelvic limb was negative. A skin biopsy was performed on the right lateral antebrachium. The area sampled was oedematous but unfortunately did not include any ulcerative lesion. Histopathological examination identified oedema without other pathological changes. Culture of the skin biopsy identified a light growth of *Enterococcus faecalis* susceptible to marbofloxacin. Secondary bacterial infection could not be ruled out. Ziehl–Neelsen staining of the biopsy was negative, providing no support for mycobacterial infection. Polymerase chain reaction (PCR) performed on oropharyngeal sampling was negative for herpesvirus and reverse-transcriptase (RT) PCR was positive for calicivirus (Idexx ®Laboratories UK). Blood RT-PCR was positive for calicivirus (Scanelis ®Laboratories France).

**Fig. 3:**
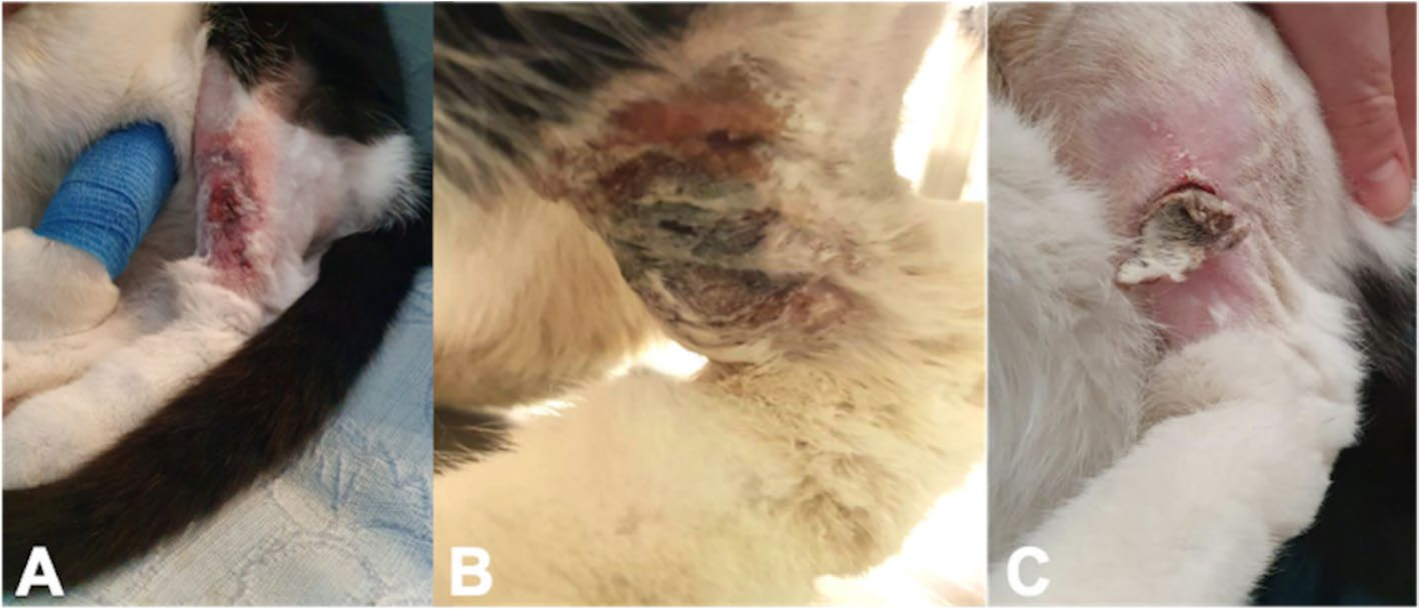
Progression of the cutaneous ulceration on presentation (**A**), on discharge after 6 days (**B**), and at home after one week (**C**)

Based on these results, a presumptive diagnosis of VS-FCV infection was made. However, given the persistent pyrexia, the necrotic appearance of the ulcerative lesions and the positive bacterial culture of the skin biopsy that had initially raised concerns of secondary bacterial infection, intravenous antimicrobial therapy (marbofloxacin, 2 mg/kg) was continued. Given the persistent pyrexia despite treatment with NSAID, intravenous corticosteroid therapy at an anti-inflammatory dose (dexamethasone, 0.1 mg/kg) was initiated after allowing a three-day wash-out period. The clinical signs progressively improved over six days and spontaneous appetite returned. Given the concern for the highly contagious nature of the virus for other cats in the hospital and in face of the progressive clinical improvement, the cat was discharged with oral corticosteroid (prednisolone, 0.5 mg/kg once daily) and antimicrobial therapy (marbofloxacin 2 mg/kg once daily). Daily follow-up over the phone were performed. The owners provided pictures to monitor the satisfactory progression of the cutaneous lesions (Fig. 3).

**Fig. 4:**
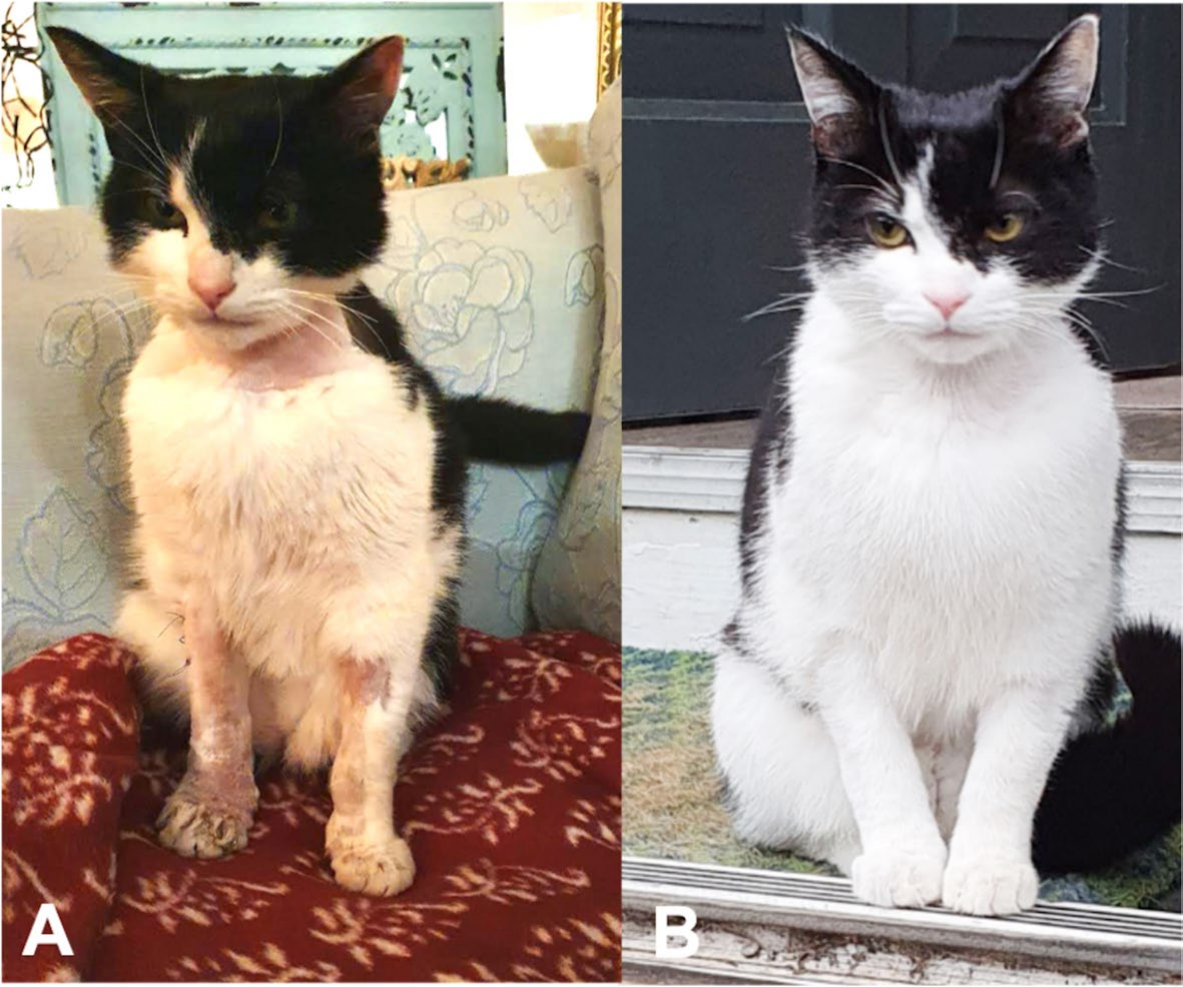
Complete resolution of the cutaneous signs 2 months after presentation (**B**) compared to immediately after discharge (**A**)

One month after discharge, the cat was reassessed. There was a complete resolution of all clinical signs (Fig. 4). Repeat blood RT-PCR was negative. Blood RT-PCR was also performed on the adopted kitten and was negative.

## Discussion and conclusions

This is the first description of a cat presented with VS-FCV infection in Ireland. The diagnosis was based on history, compatible clinical signs, exclusion of other causes, virus isolation (timely association with clinical signs), and negative virus isolation status with resolution of clinical signs.

**Fig. 5:**
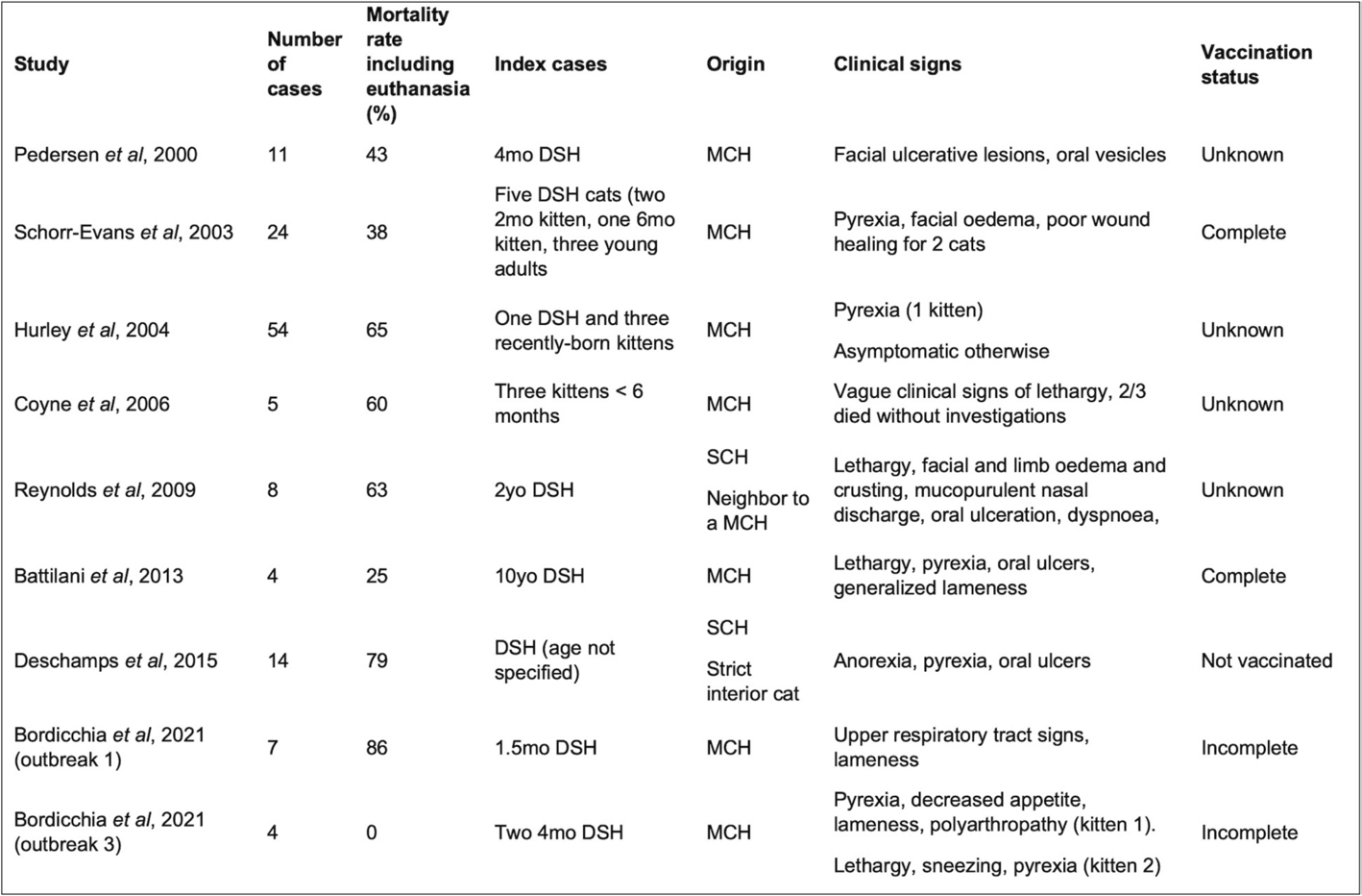
Review of the index cases in previously published cases of virulent systemic feline calicivirus outbreaks. MCH: Multi-cat household. SCH: Solo-cat household. DSH: Domestic short-hair. MO: month-old. YO: year-old

Similar to this case that was exposed to a kitten from a shelter, previous reported index cases prior to an outbreak of VS-FCV are usually young cats originating from a high-density feline household (Fig. 5) [12–19]. The clinical presentation of these index cases varies markedly, and does not seem to correlate with the efficiency of transmission or severity of disease in the resulting outbreaks. Younger cats (less than 6 months) have been shown to be more likely to display less severe clinical signs compared to adult cats [14]. As a result, it is possible that, while being infected with VS-FCV, they look apparently healthy when introduced to a new household, and thus more likely to spread infection to cats that are naïve to this strain.

It would have been interesting to investigate the suspected index kitten for calicivirus at the time of diagnosis of the affected cat. However, given the concern for the presence of a highly contagious disease, presenting the kitten to the hospital was deemed of undue risk and further investigation was not performed at that time.

The main clinical signs observed in the present case were pyrexia, pitting oedema and ulcerative lesions in accordance with previous descriptions. The most prevalent clinical signs reported to date include pyrexia (88% of the total reported cases), oedema or ulceration affecting the limbs with or without lameness (49%), oral ulceration (47%), and facial oedema and ulceration (31%). Other clinical signs less commonly reported include jaundice, gastrointestinal signs, and signs of bleeding and poorly-characterized dyspnoea [12–19, 25].

The fever is attributed to viraemia and cytokine-mediated systemic inflammation [21]. The cutaneous signs result from epidermoid and fibrinoid necrosis and related vasculitis [20]. In the present case, the skin biopsy did not identify these features and was only consistent with oedema. However, unfortunately, the area that was sampled was probably not representative, as it did not include cutaneous ulcerative lesions. Similar to the histopathology reported here, oedema with minimal inflammation has been documented on cutaneous histopathology of affected cats [14].

Clinicopathological changes associated with VS-FCV infection are variable and non-specific, including hyperbilirubinaemia, increased liver enzyme activities and thrombocytopenia [13, 14, 16, 17, 20]. Hepatocellular necrosis and pancreatitis have been documented on histological examination of affected cats and may explain the biochemical abnormalities [12–16]. Interestingly, the virus is inconsistently identified in the liver despite severe histopathological changes [1]. Sepsis can also cause icterus in cats [26] and may additionally contribute to the clinicopathological changes. The thrombocytopenia is likely consumptive, related to disseminated intravascular coagulation attributed to a combination of severe vasculopathy and marked systemic inflammation [4]. A haemorrhagic form of the disease, associated with even higher mortality, is also recognized and attributed to disseminated intravascular coagulation [13]. There were no signs of excessive haemorrhage in the present case. For this reason, investigation of a haemostatic disorder was not specifically performed.

Diagnosis relies on virus identification using real-time RT-PCR on affected organs or blood, virus isolation, or immunofluorescence [22]. Serology can demonstrate exposure but is usually not helpful given the high prevalence of non-hypervirulent calicivirus infection in cats [22]. Because of variability of the viral genome, PCR may lack sensitivity; a negative RT-PCR does not rule out FCV infection [4, 7]. There are no diagnostic assays that specifically detect VS-FCV as opposed to FCV [7]. Therefore, results in the present case could be challenged as calicivirus is common in cats and viraemia has been described in severe cases of classic oral calicivirus infection [4]. However, the combination of history of exposure to a shelter cat, compatible clinical signs, virus isolation both in blood and oropharynx at the time of emergence of clinical signs, and lack of identification of virus when clinical signs had resolved was deemed sufficient to achieve a definitive diagnosis as previously described [7]. Another diagnostic criterium is the identification of identical strains among every affected cases within an outbreak. As the suspected index case was not tested for the reasons described above, and as the cat described in the current study was the only one displaying clinical signs, this diagnostic criterium cannot be used in the current study.

Highly-contagious strains have been reported with lethal outbreaks and nosocomial infections where even the caregivers’ cats became affected [16, 18]. This is likely due to the combination of the highly contagious nature of the virus, high resistance in the environment with prolonged survival (up to 1 month) and transmission via fomites [6] along with lack of precaution while handling cats due to lack of awareness of the contagious nature of the disease [18]. Disinfection with sodium hypochlorite, isolation, barrier nursing, and contact tracing are usually useful in mitigating the environmental spread of the virus [22]. Some studies recommend that affected cats are managed at home as soon as they are clinically stable to decrease the risk of hospital spread and the development of severe cutaneous ulcerations that can develop in areas where the skin has been iatrogenically breached (eg, intravenous catheterization, blood sampling)[18]. Prolongation of hospitalization potentially increases the risk of further morbidity and mortality.

There is currently no antiviral treatment licensed against FCV, however, some compounds such as nitazoxanide have shown promising results experimentally [4, 7].

Fortunately, despite the highly-contagious nature of VS-FCV and its high mortality rate, in the case reported herein, there were no additional cases apparent, either at the referring practice or in the referral hospital. This may be due to early instigation of preventive measures or because of a non-epizootic form of the virus, which has also been reported [20, 25].

In conclusion, this is the first case of VS-FCV infection with a classic epidemiological and clinical presentation reported in Ireland to date. The cat made a full recovery and there was no lethal nosocomial outbreak. Nevertheless, it is important to raise awareness of this disease among Irish veterinary practitioners, as prompt diagnosis and appropriate preventive measures are required to limit this emerging threat for cats and veterinary practices. Virulent systemic calicivirus should be suspected in cats with pyrexia of unknown origin, cutaneous signs with limb or facial oedema and ulceration, and exposure to rescue cats from high-density households.

## Data Availability

The dataset used and analyzed during the current case study are available from the corresponding author on reasonable request.

## Abbreviations

ELISA: Enzyme-linked immunosorbent assay
FCV: Feline calicivirus
FIV: Feline immunodeficiency virus
FeLV: Feline leukaemia virus
NSAID: Non-steroidal anti-inflammatory drug
PCR: Polymerase chain reaction
RT-PCR: Reverse-transcriptase polymerase chain reaction
VS-FCV: Virulent systemic feline calicivirus
